# Unique Color Display via High‐Order Reflectance in 1D Photonic Crystals for Advanced Security Levels in Anti‐Counterfeiting Applications

**DOI:** 10.1002/advs.202508682

**Published:** 2025-08-05

**Authors:** Jae Min Bak, Yejin Kim, Min‐woo Yeo, Seo‐Hyun Jung, Hyung‐il Lee

**Affiliations:** ^1^ Department of Chemistry University of Ulsan Ulsan 44776 Republic of Korea; ^2^ Center for Advanced Specialty Chemicals Korea Research Institute of Chemical Technology (KRICT) Ulsan 44429 Republic of Korea

**Keywords:** 1D Photonic crystals, anti‐counterfeiting, high‐order photonic stop bands, humidity responsive, unique color

## Abstract

Herein, humidity‐responsive 1D photonic crystal (PC) films are developed that are capable of generating higher‐order photonic stop bands (PSBs). As a critical factor for achieving 2^nd^‐ and 3^rd^‐order PSBs, the layer thickness is modulated to achieve multi‐wavelength reflectivity within a single film. The films are fabricated using alternating layer deposition of photo‐crosslinkable poly(2‐vinylnaphthalene‐*co*‐benzophenone acrylate) (p(2VN‐*co*‐BPA)) and 52%‐quaternized poly(4‐vinylpyridine‐*co*‐benzophenone acrylate) (P4QP‐52%). Upon increasing the relative humidity (RH), the films exhibit 1^st^‐, 2^nd^‐, and 3^rd^‐order PSBs with visible color transitions across the spectrum. This behavior becomes evident as the thickness of the P4QP‐52% layer increases. Notably, for those films with relatively thick P4QP‐52% layers, the shift in the 2^nd^‐ and 3^rd^‐order PSBs is substantial enough to create composite colors, such as magenta, through the superposition of red from the 2^nd^‐order PSB and blue from the 3^rd^‐order PSB. This mechanism represents a significant advancement in PC technology and opens up new possibilities for colorimetric sensing and tunable optical materials. For practical applications, these films are further explored in anti‐counterfeiting applications, where their color transitions and hidden patterning are demonstrated. The films showed vivid color changes and distinct patterning in response to high humidity, offering potential for use in security and authentication applications.

## Graphical abstract

This study demonstrates higher‐order photonic stop bands (PSBs) in humidity‐responsive 1D photonic crystal (PC) films by modulating layer thickness, enabling 2^nd^‐ and 3^rd^‐order PSBs with multi‐wavelength reflectivity. Alternating layers of p(2VN‐*co*‐BPA) and P4QP‐52% produce distinct color shifts and composite colors, advancing PC applications in colorimetric anti‐counterfeiting labels.

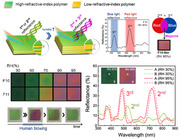

## 1 Introduction

Photonic crystals (PCs) have garnered significant attention due to their ability to manipulate light across various wavelengths through periodic dielectric structures. These materials have potential applications such as sensing,^[^ ^1^, ^2^, ^3^, ^4^ ^]^ display technologies,^[^ ^5^, ^6^, ^7^, ^8^ ^]^ and optical filtering^[^ ^9^, ^10^ ^]^ due to their unique optical properties, including photonic stop bands (PSBs) wherein certain wavelengths of light are reflected. Stimuli‐responsive 1D PCs have attracted increasing interest due to their unique ability to manipulate light by adjusting their PSBs in response to various external stimuli, such as temperature,^[^ ^11^, ^12^, ^13^, ^14^ ^]^ pH,^[^ ^15^, ^16^ ^]^ and humidity.^[^ ^17^, ^18^, ^19^ ^]^ These materials can alter their structural color dynamically, making them valuable for a broad range of applications, including environmental sensors,^[^ ^20^, ^21^, ^22^, ^23^ ^]^ optical devices,^[^ ^24^, ^25^, ^26^, ^27^, ^28^ ^]^ and display technologies^[^ ^29^, ^30^, ^31^, ^32^ ^]^​. In particular, the ability to tune the optical response across different wavelengths allows for the development of sensors with optical readouts that are visible to the naked eye. Humidity‐responsive PCs offer a promising platform for color tuning, as they undergo structural changes in response to varying relative humidity (RH) levels, leading to shifts in their reflection wavelengths. These shifts, particularly for higher‐order PSBs, can result in the generation of composite colors that arise from the combination of multiple reflected wavelengths. Such tunability could be crucial in developing advanced optical devices with dynamically adjustable color outputs.

As advancements in material fabrication techniques have enabled more precise control over the PC structures, there has been increasing interest in exploring higher‐order PSBs. However, while significant research has emerged on higher‐order reflections in PCs in recent years, the application of 2^nd^‐ and 3^rd^‐order PSBs in response to external stimuli, particularly in 1D PCs and stimuli‐responsive systems, remains relatively underexplored compared to 1^st^‐order responses. This is partly due to the technical challenges associated with generating and controlling higher‐order reflections in structured materials, particularly under external stimuli such as humidity,^[^ ^33^ ^]^ temperature,^[^ ^34^, ^35^, ^36^ ^]^ or electric fields.^[^ ^37^, ^38^ ^]^ As a result, many researchers have focused on 1^st^‐order responses due to their immediate applicability and simpler experimental setups.

In the present study, higher‐order PSBs are implemented by varying the layer thickness within the 1D PC system, as this is a critical factor for achieving 2^nd^‐ and 3^rd^‐order PSBs. The ability to precisely tune the thickness of individual layers allows for enhanced control over the interaction between light and the crystal structure. This control is pivotal in extending the functionality of the 1D PCs beyond 1^st^‐order reflections, thereby enabling the generation of 2^nd^‐ and 3^rd^‐order PSBs. By carefully adjusting the refractive index contrast and layer thickness, it is demonstrated that the 1D PCs can exhibit multi‐wavelength reflectivity upon humidity variation. This approach underscores the importance of thickness as a key design variable for achieving complex optical responses in 1D systems. In detail, the humidity‐responsive 1D PC films are fabricated via the alternating layer deposition of photo‐crosslinkable poly(2‐vinylnaphthalene‐*co*‐benzophenone acrylate) (p(2VN‐*co*‐BPA)) and 52%‐quaternized poly(4‐vinylpyridine‐*co*‐benzophenone acrylate) (P4QP‐52%). While the concentration of p(2VN‐*co*‐BPA) is fixed at 2.5%, that of P4QP‐52% is increased in increments of 0.3%, from 1.7% to 4.7%, thus resulting in a total of 11 samples with various layer thicknesses. These films initially display vivid color transitions as the RH is increased from 30% to 95%. However, as the thickness of the P4QP‐52% layer increases, 1^st^‐, 2^nd^‐, and 3^rd^‐order PSBs emerge with distinct color transitions in the visible spectrum range according to the variation in RH. Moreover, a further increase in the thickness of the P4QP‐52% layer leads to the creation of composite colors through the superposition of red from the 2^nd^‐order PSB and blue from the 3^rd^‐order PSB. Additionally, this unique color generation mechanism was demonstrated in anti‐counterfeiting films for practical applications. Two hidden patterned films with high humidity responsiveness were fabricated. When blown upon by human breath, the films revealed distinct hidden flower‐ and leaf‐patterns. This work represents a significant step toward the development of security and authentication systems, as well as multi‐wavelength, tunable photonic systems driven by environmental stimuli.

## 2 Results and Discussion

**Figure 1:**
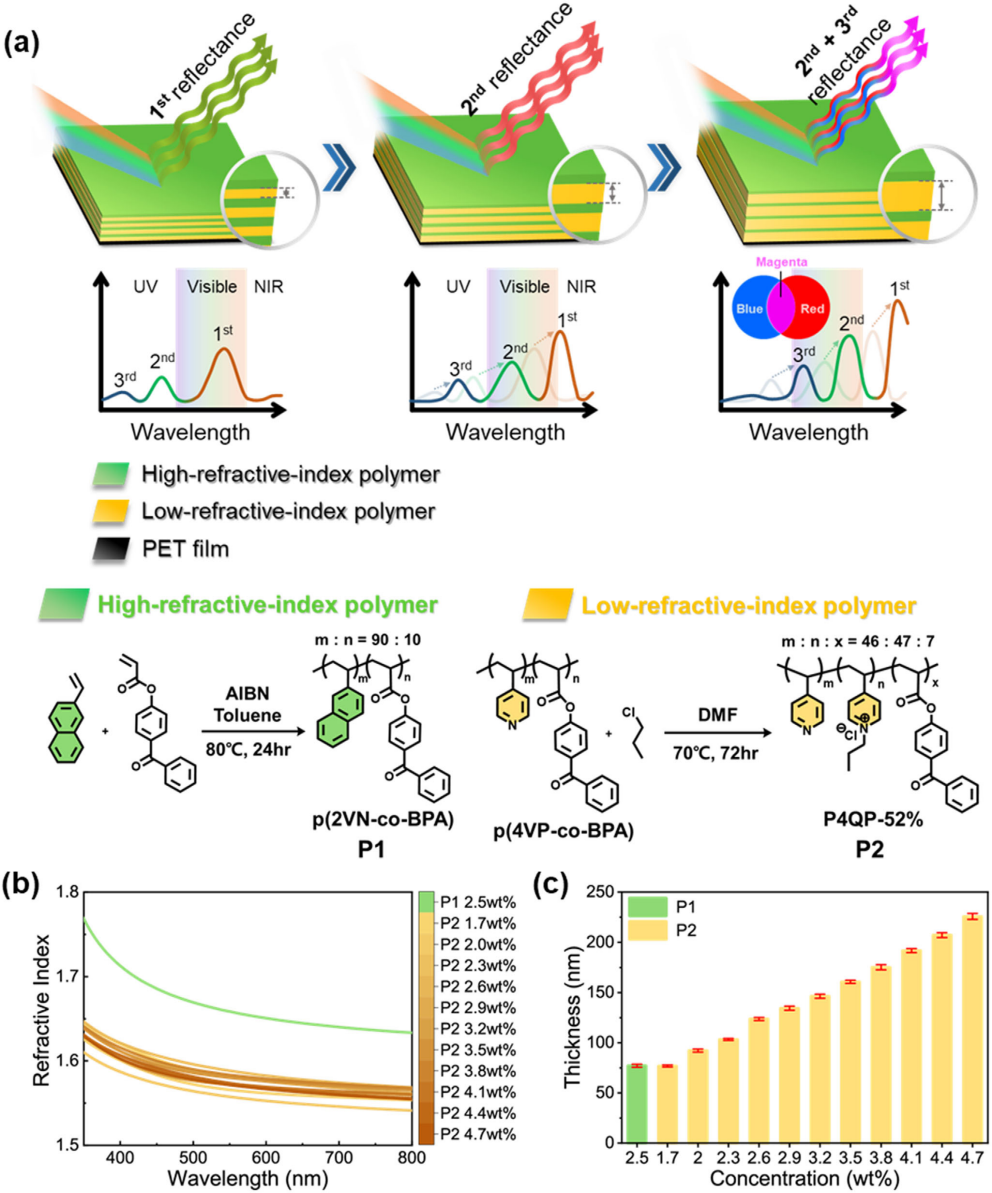
a) Schematic diagrams of the humidity‐responsive 1D PC films capable of generating higher‐order PSBs and creating composite colors within a single film (top row), along with the syntheses of the high‐refractive‐index P(2VN‐*co*‐BPA) (P1) and low‐refractive‐index 52%‐quaternized P(4VP‐*co*‐BPA) (P2) (bottom row). b and c) The refractive indices (b) and monolayer thicknesses (c) of the spin‐coated P1 film and the various P2 films on silicon wafers.

To demonstrate the appearance of humidity‐induced high‐order PSBs and new reflection colors within a single film, a PC structure was designed wherein the reflection wavelength can shift to longer wavelengths with high reflectivity upon an increase in humidity, as shown in **Figure** **1**a. As the humidity increases, the 1^st^‐order reflected wavelength shifts to the near‐infrared (NIR) region, while the 2^nd^‐ and 3^rd^‐order reflected wavelengths appear in the visible spectrum. This results in the combination of two reflected wavelengths, thereby producing a composite color. To achieve the high‐order reflectance, the high‐ and low‐refractive index photo‐crosslinkable copolymers P1 and p(4VP‐*co*‐BPA) were synthesized via FRP with 8–10 mol% BPA to ensure sufficient crosslinking density, as detailed in the Experimental section. In addition, the p(4VP‐*co*‐BPA) was treated with 1‐chloropropane to obtain P2 with a degree of quaternization (DQ) of 52%. The successful synthesis of these polymers was confirmed by ^1^H NMR spectroscopy and GPC analysis (Figures S1,S2, Supporting Information).

The refractive indices and monolayer thicknesses of the high‐refractive‐index (P1) and various low‐refractive‐index (P2) monolayer films are revealed by the ellipsometry results in Figure 1b,c. Thus, at 633 nm, the refractive index of P1 is found to be 1.64, while that of P2 ranges from 1.56 to 1.59 depending on its concentration in propanol (Figure 1b). Thus, the difference in refractive index between P1 and P2 is between 0.05 and 0.08. Meanwhile, the thickness of P1 is 77 nm, and that of P2 gradually increases from 77 to 226 nm with increasing concentration (Figure 1c).

For 1D PCs, the realization of higher‐order PSBs primarily relies on the ease of controlling the film thickness. While several factors, such as spin‐coating speed and solvent type also play important roles, the most critical factor in determining film thickness is the concentration of the polymer solution. The relationship between concentration and thickness is crucial, as it directly influences the tunability of the PC's optical properties, including the ability to generate higher‐order PSBs. The concentration of the polymer solution dictates the maximum achievable film thickness, which in turn defines the range of PSBs. Thus, the results in Figure 1b suggest an upper limit for the achievable thickness, corresponding to a critical point determined by the solution concentration. Beyond this point, increasing the concentration leads to a decline in control over the optical performance and uniformity of the film. Thus, this critical thickness represents the threshold for the practical realization of higher‐order PSBs in 1D PCs, highlighting the vital role of solution concentration in enabling complex optical responses. Specifically, an examination of Figure 1b suggests an optimum P2 concentration of 4.7 wt.%, giving a thickness of 226 nm.

**Figure 2:**
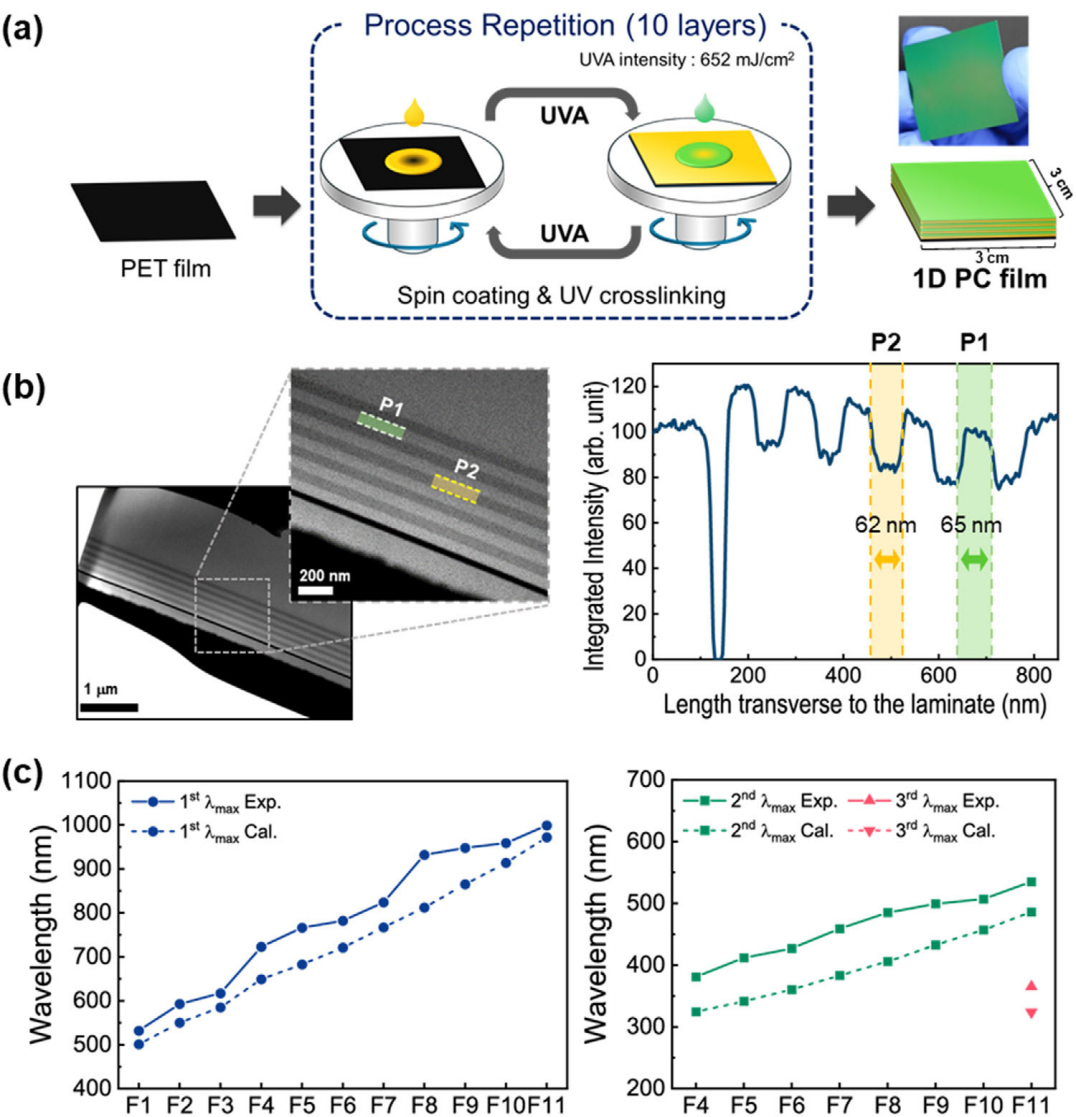
a) A schematic diagram showing the fabrication process of the humidity‐responsive 1D PC multilayer films based on alternate layers of high‐ and low‐refractive‐index polymers. b) The cross‐sectional STEM images (left) and corresponding intensity spectra (right) of the 10‐layer stack (F1) c) Plots of the experimental and calculated 1^st^‐order (left) and 2^nd^‐ and 3^rd^‐order (right) λ_max_ values at 30% RH.

Humidity‐responsive 1D PC multilayers were fabricated by alternately depositing P1 and P2 onto a black PET film, as detailed in the Experimental section and shown schematically in **Figure** **2**a. The STEM images and corresponding depth intensity profile of the 10‐layered film (F1) are presented in Figure 2b. Here, a well‐ordered parallel lamellar morphology is observed, with the P1 layers (bright bands) exhibiting a thickness of 62 nm, and the P2 layers (dark bands) exhibiting a thickness of 65 nm. The average thickness of five P1 layers is 63 nm (with a standard deviation of 2.0 nm), and that of five P2 layers is 62 nm (with a standard deviation of 1.2 nm).

Based on the above thickness and refractive index measurements, the high‐order theoretical λ_max_ is calculated using Equations (1) and (2):

(1) $$\lambda_{max}=2\left(n_{l}d_{l}+n_{h}d_{h}\right)$$

(2) $$\lambda_{n}=\frac{\lambda_{max}}{n}\;\left(n=1st,2nd,3rd,etc.\right)$$

where λ_max_ is the 1^st^‐order reflected wavelength, *n_l_* and *n_h_* are the refractive indices of the respective low‐ and high‐refractive index materials (*n_h_* > *n_l_*), *d_l_* and *d_h_* are the thicknesses of the low‐ and high‐refractive index materials, λ_n_ represents the order of reflected wavelength.

The theoretical 1^st^‐, 2^nd^‐, and 3^rd^‐order reflected wavelength values are presented, along with the experimental values, in Figures 2c and S3 (Supporting Information). Thus, as the film thickness increases from F1 to F11, the experimental 1^st^‐order λ_max_ values increase from 532 to 999 nm, while the corresponding theoretical values are 501 and 972 nm, respectively. For the F4 film, the experimental and theoretical 2^nd^ order λ_max_ values are 325 and 381 nm, respectively, while those of the F11 film are 486 and 535 nm, respectively. Meanwhile, the 3^rd^ order λ_max_ is observed only in F11, with an experimental value of 365 nm and a theoretical value of 324 nm. These results indicate good agreement between the experimental and theoretical 1^st^, 2^nd^, and 3^rd^ order λ_max_ values of the F1–F11 films (Table 1).

**Table 1:** Theoretical and experimental 1^st^‐, 2^nd^‐, and 3^rd^‐order PSBs of eleven films at 30% RH.

Sample	PSBs at humidity 30%					
	1^st^ order		2^nd^ order		3^rd^ order	
	Cal.	Exp.	Cal.	Exp.	Cal.	Exp.
F1	501	532	251	‐	167	‐
F2	550	593	275	‐	183	‐
F3	585	617	292	‐	195	‐
F4	649	723	325	381	216	‐
F5	683	766	342	412	228	‐
F6	721	782	361	427	240	‐
F7	767	824	383	459	256	‐
F8	812	932	406	485	271	‐
F9	865	948	432	499	288	‐
F10	914	959	457	507	305	‐
F11	972	999	486	535	324	365

**Figure 3:**
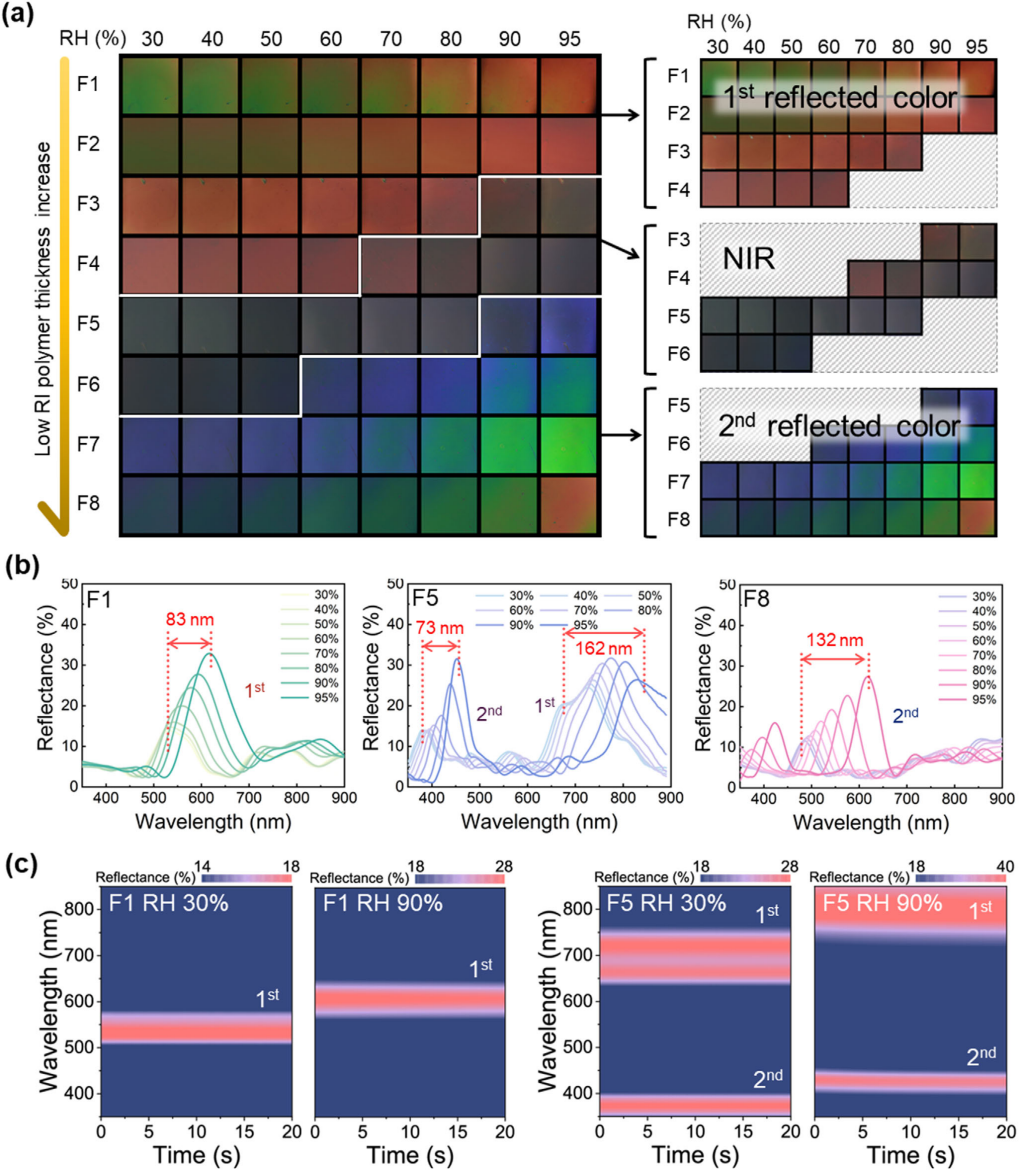
a) Photographic images of samples F1 to F8 showing the color changes when exposed to various RH levels (30%–95%). b) The reflectance spectra of samples F1, F5, and F8 at various RH values. c) The DRS results for (left) the F1sample at 30% and 90% RH, and (right) the F5 sample at 30% and 90% RH.

The humidity‐induced behaviors of the PC films are revealed by the color differentiation maps, in situ UV–vis reflectance spectra, and DRS results in **Figure** **3**. Here, samples F1–F4 clearly exhibit distinct color transitions relating to 1^st^‐order reflection as the RH increases from 30% to 95% at a fixed temperature of 25 °C. Specifically, F1 changes from green to red, F2 from yellow to red, F3 from orange to near‐infrared (NIR), and F4 from red to NIR. By contrast, samples F5–F8 display color transitions associated with a 2^nd^‐order reflection. For instance, while F5 is initially colorless at 30% RH due to the 1^st^‐order reflection located in the NIR region, it becomes blue at 95% RH due to the appearance of the 2^nd^‐order reflection. Meanwhile, F6 remains colorless at 30% to 50% RH, and then transitions from blue at 69% RH to green at 90–95% RH. For F7 and F8, the initial colors originate from the 2^nd^‐order reflection at 30% RH, so that these films transition from blue to green and from green to orange, respectively, as the RH increases from 30 to 95%.

The reflectance spectra of F1, F5, and F8 are shown in Figure 3b, while those of F2–F4 are presented in Figure S4 (Supporting Information). From these results, the 1^st^‐order PSB is seen to shift by 83, 138, 140, and 149 nm for F1, F2, F3, and F4, respectively, as the RH increases from 30 to 95%. Meanwhile, the 2^nd^‐order PSB first appears in F2 at a high RH of 95% and is shifted into the visible region at RH values of 80 and 95 for F3 and F4 (Figure S4, Supporting Information). For F5 (middle panel, Figure 3b), the 1^st^‐order wavelength shifts to the NIR region with increasing RH, while the 2^nd^‐order PSB shifts by 73 nm, from 380 to 453 nm. For F6 and F7 (Figure S4, Supporting Information), the 2^nd^‐order PSB shifts by 91 nm (from 427 to 518 nm) and 103 nm (from 454 to 557 nm), respectively. For F8 (right‐hand panel, Figure 3b), the 2^nd^‐order PSB shifts by 132 nm, from 485 to 617 nm. The reflectivity of F1–F8 gradually increases with the RH value due to the penetration of water molecules (n = 1.33) into the quaternized low refractive‐index layers, which results in swelling and, hence, a decrease in the refractive index.

In the case of F1, the DRS spectrum in Figure 3c (left‐hand panels) reveals a shift in the 1^st^‐order PSB from 534 nm at 30% RH to 614 nm at 90% RH. In the case of F5, however, two PSBs are observed, with the 1^st^‐order PSB shifting from 667 nm at 30% RH to 829 nm at 90% RH, and the 2^nd^‐order PSB shifting from 380 to 453 nm, respectively. Notably, the reflectance wavelength remains stable over time once the humidity level is constant, thereby indicating that the wavelength shifts are driven solely by changes in humidity, not by the passage of time. These results demonstrate that the DRS results provide a clear indication of how the PSBs shift to longer wavelengths with increasing humidity, thereby reinforcing the significant role of moisture‐induced changes in refractive index and layer thickness in modulating the optical properties of the PC films. In addition, ensuring film stability during repeated humidity sensing is crucial for practical applications. Therefore, the specific shift in the PSB for F1 was monitored across 20 cycles of RH variation from 30% to 95%. As shown in Figure S5 (Supporting Information), the results indicated excellent durability.

**Figure 4:**
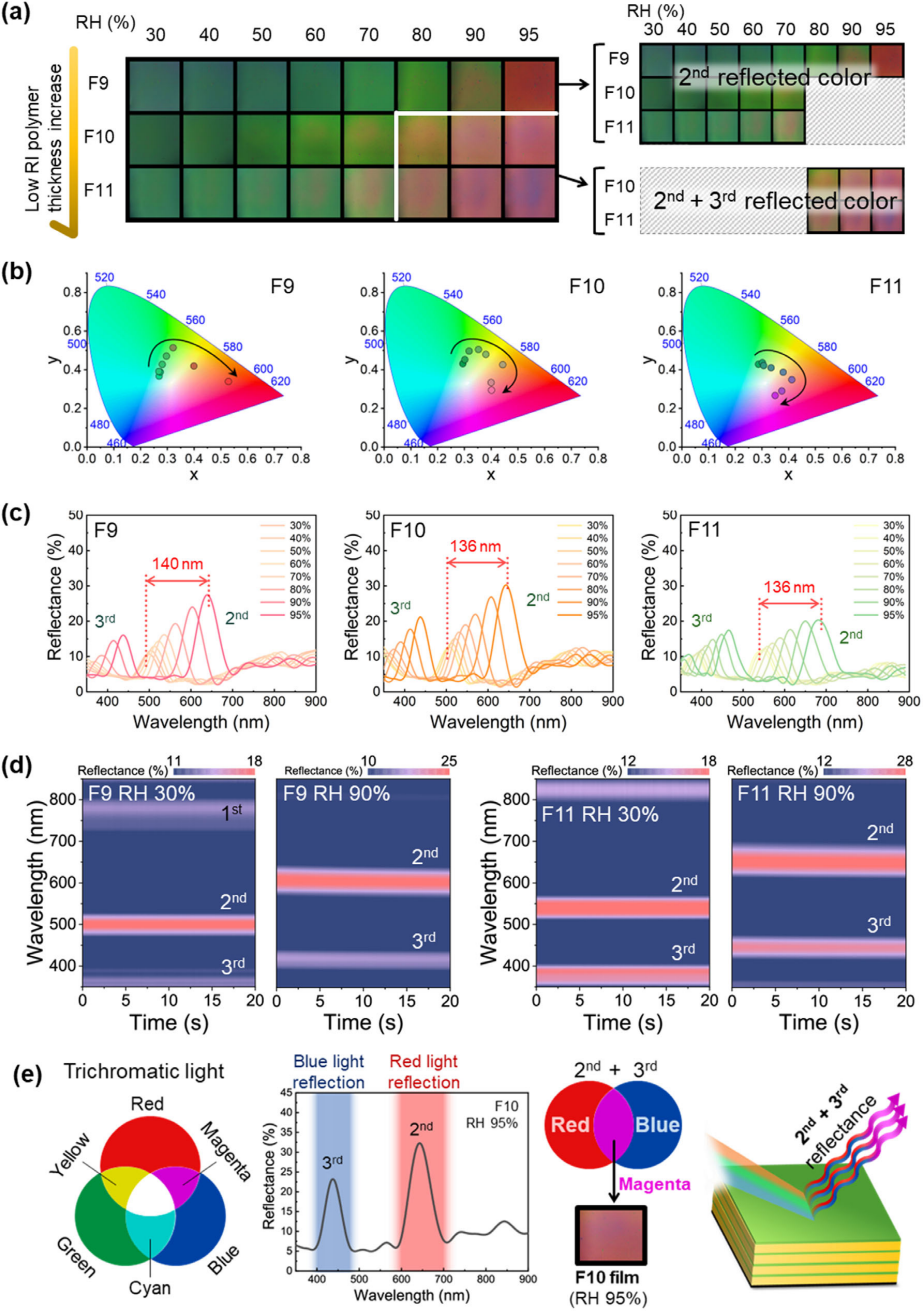
a) Photographic images of F9–F11 showing the color changes at RH levels of 30–95%. b) The chromaticity diagrams (CIE 1931) of F9 to F11. c) The reflectance spectra of F9 to F11 at various RHs. d) The DRS results for the F9 (left) and F11 (right) at 30 and 90% RH. e) A schematic illustration of the composite color generation mechanism due to the combination of 2^nd^‐ and 3^rd^‐order reflections.

The composite color generation due to the combination of 2^nd^‐ and 3^rd^‐order reflections in the 1D PC multilayer samples F9–F11 at RH values of 30–90% and a fixed temperature of 25 °C are investigated in **Figure** **4**. Thus, in Figure 4a, the F9 and F10 films exhibit color transitions from green to red and from green to orange, respectively, as the RH increases up to 90% due to 2^nd^‐order reflection. However, a new color, namely magenta, appears at 95% RH. Similarly, for F11, a color transition from green to orange is observed at RH values of up to 80%, and a new composite color appears above 90% RH. These color transitions are confirmed by the CIE 1931 chromaticity coordinates of the F9, F10, and F11 films in Figures 4b and S6, and Table S2 (Supporting Information). Here, F9 exhibits a color transition from green to red, whereas F10 and F11 each show a color transition from green to magenta, as the RH increases from 30 to 95%. This analysis further highlights how the combination of 2^nd^‐ and 3^rd^‐order reflections results in a shift from green to more complex hues, such as magenta, as the RH increases. This provides valuable insights into the tunability of these multilayer films for optical applications.

These new colors are further revealed by the UV–vis reflectance spectra in Figure 4c. Here, F9 exhibits significant shifts in its PSBs as the RH increases from 85% to 95%. Specifically, the 2^nd^‐order PSB is shifted by 140 nm, from 499 to 639 nm, while the 3^rd^‐order PSB is shifted by 61 nm, from 369 nm to 430 nm. Both F10 and F11 display 2^nd^‐ and 3^rd^‐order PSBs within the visible region under high RH conditions. For F10, the 2^nd^‐order PSB is shifted by 136 nm, from 507 to 643 nm, as the RH increases from 30 to 95%, whereas the 3^rd^‐order PSB is shifted by 66 nm, from 372 to 438 nm. Similarly, for F11, the 2^nd^‐order PSB is shifted by 136 nm, from 546 to 682 nm, and the 3^rd^‐order PSB is shifted by 77 nm, from 389 to 466 nm.

The time‐dependent DRS analyses of the 2^nd^‐ and 3^rd^‐order PSBs for the F9 and F11 samples at 30 and 90% RH are presented in Figure 4d. Thus, at 30% RH, the F9 exhibits a 2^nd^‐order PSB at 499 nm and a 3^rd^‐order PSB at 369 nm. At 90% RH, however, the 2^nd^‐order PSB has shifted to 607 nm, and the 3^rd^‐order PSB has shifted to 411 nm. In the case of F11, the 2^nd^‐order PSB shifts from 546 nm at 30% RH to 682 nm at 90% RH, while the 3^rd^‐order PSB shifts from 389 to 466 nm.

Finally, the composite color mechanism of the humidity‐induced high‐order PC is shown schematically in Figure 4e. Here, white light is shown as trichromatic blue, green, and red (left), where the magenta color exhibited by F10 and F11 above 90% RH is seen to be a composite color. This is obtained by combining the 2^nd^‐ and 3^rd^‐order reflections. Specifically, for F10 at 95% RH, the 2^nd^‐order PSB reflects the red region, while the 3^rd^‐order PSB reflects the blue region, thus resulting in the composite color of magenta. This is the first time that two reflection wavelengths have been realized in a single film based on higher‐order reflection wavelengths induced by humidity.

**Figure 5:**
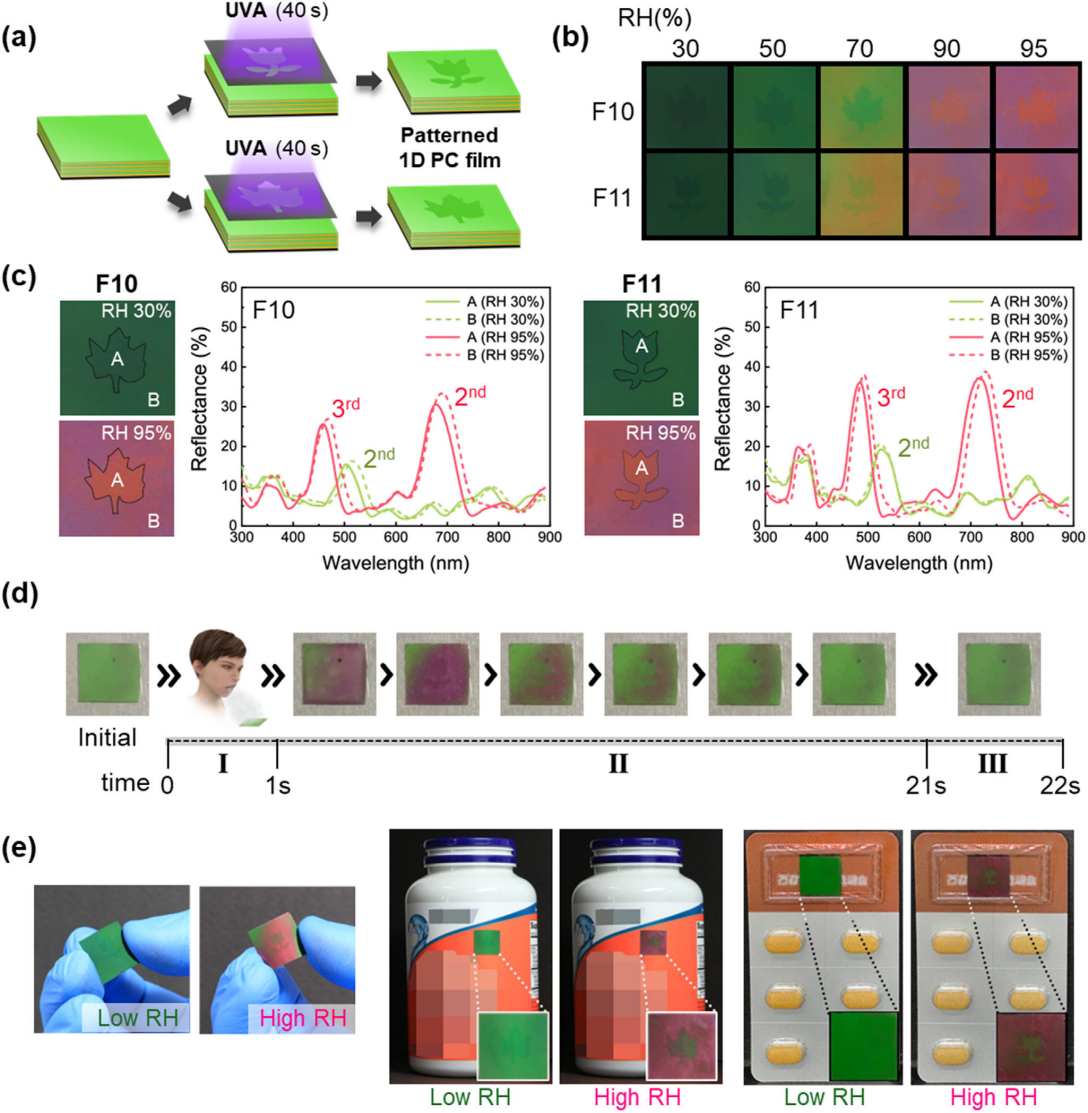
a) A schematic diagram showing the patterning process of two different patterned films. b) Photographic images of the patterned F10 and F11 films under various RH values (30 to 95%). c) Photographic images and reflectance spectra of the patterned interior A and background B at RH values of 30% and 95% d) Photographic images of the color transitions of the leaf‐ and flower‐patterned film due to human breath (blowing). e) Photographs of the flexible and patterned 1D photonic crystal multilayer films transferred onto plastic bottles and medicine packaging as anti‐counterfeiting applications.

For the practical implementation of high humidity‐responsive color transitions in anti‐counterfeiting labels, two different patterned films were fabricated, as illustrated in **Figure** **5**a. Flower‐ and leaf‐patterned masks were applied to the pre‐fabricated F10 and F11 films, which were then exposed to UV light for 40 s. Patterning occurred only in the areas exposed to UV light, and exposure for longer than 40 s resulted in damage to the films. The patterned shapes on the hidden film were created solely through the UV exposure process, without the need for any additional coating steps. This method utilizes further crosslinking of the residual benzophenone moiety, which increases the crosslinking.

The colorimetric responses of the two patterned films to humidity are shown in the color differentiation maps in Figure 5b. Both films exhibited distinct color transitions as the RH increased from 30% to 95%. At 30% RH, the patterned shapes were not visible on the films. However, when the RH exceeded 50%, the patterned shapes began to emerge along with the color transition. At 95% RH, the color of each patterned film became vivid magenta color (background) and red (interior), with clearly defined patterns.

The reflectance spectra of the internal pattern areas (labeled A in the photographic images) and background areas (labeled B) for both patterned films at RH values of 30% and 95% are presented in Figure 5c. At 30% RH, the leaf‐patterned film exhibited a 2^nd^‐order PSB at 503 nm for the interior and 514 nm for the background. However, at 95% RH, the 2^nd^‐order PSB of the leaf‐patterned film shifted to 679 nm (interior) and 691 nm (background). Additionally, the 3^rd^‐order PSB was observed at 458 nm (interior) and 467 nm (background).For the flower‐patterned film, at 30% RH, the 2^nd^‐order PSB appeared at 523 nm for the interior and 525 nm for the background. At 95% RH, the 2^nd^‐order PSB of the flower‐patterned film shifted to 717 nm (interior) and 728 nm (background). The 3^rd^‐order PSB was observed at 484 nm (interior) and 492 nm (background). These shifts in the 2^nd^‐ and 3^rd^‐order PSBs highlight the humidity‐dependent changes in the optical properties of the films. The color transitions from RH 30% to RH 95% for both the leaf (A and B) and flower (A and B) patterns are shown in the corresponding photographic images, along with the reflectance spectra indicating the changes in the films' optical characteristics as the RH increases.

The high humidity sensitivity of the two patterned films for anti‐counterfeiting applications is presented in Figure 5d and further illustrated in Video S1 (Supporting Information). When a person blew onto the film (I), the color changed from green to magenta, revealing a distinct hidden flower‐patterned shape (II). When the blowing stops, the film returned to its original color (III). Furthermore, as the films are coated onto a black PET substrate, they can be applied to various items or materials (e.g., plastic bottles or medicine packages) for anti‐counterfeiting purposes. This is demonstrated in the photographs in Figure 5e, which show that the vivid color transition and hidden shape occurs exclusively in a high‐humidity environment. These films can be readily applied to a wide range of materials.

## 3 Conclusion 

This study demonstrated the successful fabrication and characterization of humidity‐responsive 1D photonic crystals (PCs) that exhibit 1^st^‐, 2^nd^‐, and 3^rd^‐order photonic stop bands (PSBs). By systematically varying the refractive index contrast and layer thickness, significant shifts in the PSBs were observed in response to changes in relative humidity (RH), ranging from 30% to 95%. The emergence of higher‐order PSBs, particularly under high RH conditions, resulted in distinct color transitions and the generation of composite colors within the visible spectrum. Notably, the sample designated as F10 showed shifts in the 2^nd^‐ and 3^rd^‐order PSBs from 507 to 643 nm and 400 to 440 nm, respectively. Furthermore, these films were explored for practical applications in anti‐counterfeiting labels, where their color transitions and hidden patterns were demonstrated. This work marks a significant advancement in the development of multi‐wavelength, adjustable photonic systems that are responsive to environmental stimuli.

## Conflict of Interest

The authors declare no conflict of interest.

## Author Contributions

Jae Min Bak and Yejin Kim contributed equally to this work.

## Supporting information



Supporting Information

Supplemental Video 1

## Data Availability

The data that support the findings of this study are available from the corresponding author upon reasonable request.
