# Supplementary material for: Unique Color Display via High‐Order Reflectance in 1D Photonic Crystals for Advanced Security Levels in Anti‐Counterfeiting Applications
Source: Adv Sci (Weinh). 2025 Aug 5;12(37):e08682. doi: 10.1002/advs.202508682 (PMC12499487; doi:10.1002/advs.202508682)
Supplement: Supplementary file 1 — Supporting Information [file ADVS-12-e08682-s001.docx]

Unique Color Display Driven by High-Order Reflectance in 1-Dimensional Photonic Crystals for Advanced Security Levels in Anti-Counterfeiting Applications

Jae Min Bak^a,1^, Yejin Kim^b,1^, Min-woo Yeo^a^, Seo-Hyun Jung^b^*, Hyung-il Lee^a^*

^a^Department of Chemistry, University of Ulsan, Ulsan 44776, Republic of Korea

^b^Center for Advanced Specialty Chemicals, Korea Research Institute of Chemical Technology (KRICT), Ulsan 44429, Republic of Korea

**Table of Contents**

1. Experimental Section 2

2. Supplementary Figures5

**Table S1**. The basic details of the eleven 1D PC film samples prepared with various thicknesses)5

**Figure S1.** ^1^H NMR spectra for synthesis of 4-Benzophenyl acrylate, p(2VN-*co*-BPA) (P1), p(4VP-*co*-BPA), and Quaternized p(4VP-*co*-BPA) (P2).6

**Figure S2.** DMF GPC trace of (a) p(2VN-*co*-BPA), and (b) p(4VP-*co*-BPA).7

**Figure S3.** Comparing the simulated theoretical reflectance to the experimental reflectance P2 2.0, 2.6 and 4.7 wt%.(under humidity 30%)7

**Figure S4.** The reflectance spectra of samples F1 to F8 at various RH values.8

**Figure S5.** Reflectance spectra changes of sample F1 during 20 alternating cycles at various RH values.8

**Table S2**. The RGB values of F1 to F11 at various RH values..9

**Figure S6.** The chromaticity diagrams (CIE 1931) of F1 to F11 at various RH values.9

1. Experimental Section

1.1. Materials

4-Hydroxybenzophenone (98.0%), 4-vinylpyridine (95%), and 2-vinylnaphtalene (97.0%) were obtained from Alfa Aesar. Acryloyl chloride (98.0%) was obtained from Tokyo Chemical Industry (TCI) Co., Ltd. and was employed without further purification. 2,2’-Azobisisobutyronitrile (AIBN, recrystallized from methanol, 99.0%), triethylamine (TEA, 99.0%), tetrahydrofuran (THF, 99.0%), N,N-dimethylformamide (DMF, 99.0%), dichloromethane (DCM, 99.5%), toluene (99.5%), 1-propanol (anhydrous, 99.5%), chlorobenzene (99.0%), and 1-chloropropane (98.0%) were purchased from Sigma-Aldrich.

1.2. Characterization

^1^H Nuclear magnetic resonance (NMR) experiments were conducted in chloroform-*d* (CDCl_3_), and methanol-*d*_4_ (MeOD) on a Bruker Avance III 400 MHz spectrometer at the total-period analysis center for Ulsan chemical industry of Korea Basic Science Institute (KBSI). The apparent molecular weights and molecular weight distributions of the polymers were measured by gel permeation chromatography (GPC, 1260 Infinity, Agilent) using a poly(methyl methacrylate (PMMA) standard with DMF as the eluent at 30 °C and a flow rate of 1.00 mL/min. The refractive index of each single-layer polymer film on a silicon wafer was determined by ellipsometry (HORIBA Scientific, UVISEL). After spin-coating the alternate polymer layers on black polyethylene terephthalate (PET) plates, their colorimetric responses to changes in humidity were examined using a humidity test chamber. The spectral reflectance was observed using an ultraviolet-visible (UV−vis) spectrometer (USB4000, OceanOptics Inc.), and the time-dependent dynamic reflectance spectra (DRS) were obtained. Cross-sectional images of the polymer laminates were then acquired via bright-field, four-channel scanning transmission electron microscopy (STEM; GEMINISEM 500, ZEISS) at an acceleration voltage of 30 kV. However, TEM at 200 kV failed to produce a sufficient contrast difference between the two alternating polymer layers in the laminate. 1D photonic crystal (PC) multilayers on black plates were placed in a humidity and temperature test chamber(S-THSC31R1,SERIMA), where the UV-vis reflectance spectra(USB4000, OceanOptics Inc.) were recorded in situ. The temperature inside the chamber was maintained at a constant 25°C, and measurements were taken as the humidity increased from 30% to 95%.

1.3. Syntheses

1.3.1. 4-Benzophenyl acrylate (BPA).

4-hydroxybenzophenone (15 g, 75.7 mmol) and TEA (10.55 mL, 75.7 mmol) were dissolved in DCM (450 mL) in a round-bottomed flask. The reaction mixture was stirred in an ice bath, after which a solution of acryloyl chloride (7.34 mL, 90.8 mmol) in DCM (50 mL) was added dropwise. The reaction was allowed to proceed for 30 min at 0 °C with constant stirring, after which the mixture was allowed to cool to room temperature and stirring was continued overnight. The product was then washed with 5% sodium bicarbonate, brine, and distilled water to remove the unreacted materials, dried with magnesium sulfate, then filtered and evaporated. Finally, the BPA product was recrystallized to obtain a white powder (13 g, 87% yield). ^1^H NMR (400 MHz, CDCl_3_: δ [ppm]): 7.25–7.95 (m, 9H –C_6_*H*_4_ (C=O)–C_6_*H*_5_); 6.66 (dd, 1H, CH_2_–C*H*–(C=O)); 6.04–6.40 (dd, 2H, –C*H*_2_–CH–(C=O)).

1.3.2. p(2VN-*co*-BPA)

The p(2VN-*co*-BPA) copolymer (designated as **P1**) was synthesized via free-radical polymerization (FRP) using AIBN as a free radical initiator. In detail, 4-vinylnaphthalene (5.0 g, 32.4 mmol), BPA (0.9036 g, 3.6 mmol), AIBN (0.012 g, 0.0731 mmol), and toluene (15 mL) were added to a Schlenk flask. After purging the solution with pure argon (Ar) gas for 15 min, polymerization was conducted at 80 °C in an oil bath for 24 h. The polymerization was then stopped by exposure to air and precipitated twice using methanol. The polymer was filtered and dried overnight under vacuum at 30 °C to obtain the product as a white solid (3.6 g, 61% yield; M_n_ = 31,900 g/mol, M_w_/M_n_ = 1.77, n ≈ 1.68). ^1^H NMR (400 MHz, CDCl_3_: δ [ppm]): 6.19–7.75 (16H, C_10_*H*_7_, –C_6_*H*_4_–(C=O)–C_6_*H*_5_); 1.15–2.38 (6H, –C*H*_2_–C*H*–C*H*_2_–C*H*–).

1.3.3. p(4VP-*co*-BPA)

4-Vinylpyridine (10.24 mL, 95 mmol), BPA (2.64 g, 10.56 mmol), and AIBN (0.034 g, 0.21 mmol) were dissolved in DMF (30 mL) and added to a Schlenk flask. The solution was purged with pure Ar gas for 15 min and then polymerized at 60 °C in an oil bath for 14 h, after which the reaction was stopped by exposing it to air. The product was precipitated twice using diethyl ether and dried overnight under vacuum at 30 °C to obtain a white solid (7.3 g, 62% yield; M_n_ = 105,700 g/mol, M_n_/M_w_ = 2.61, n ≈ 1.58). ^1^H NMR (400 MHz, MeOD: δ [ppm]): 7.94–8.46, 6.47–7.09 (4H, –C–C*H*–C*H*–N–C*H*–C*H*); 7.39–7.86 (9H, –C_6_*H*_4_–(C=O)–C_6_*H*_5_); 1.31–2.65 (6H, –C*H*_2_–C*H*–C*H*_2_–C*H*–).

1.3.4. Quaternized p(4VP-*co*-BPA)

To obtain the quaternized p(4VP-co-BPA) (designated as **P2**), p(4VP-*co-*BPA) (3.0 g) and excess 1-chloropropane (10 mL) were dissolved in a round-bottomed flask containing DMF (30 mL). The solution was purged with Ar gas for 10 min and then quaternized at 70 °C in an oil bath for 5 days. The polymer was then precipitated twice in diethyl ether, filtered, and dried under vacuum at 30 °C to yield a brown solid (3.0 g). ^1^H NMR (400 MHz, MeOD: δ [ppm]): 8.46–9.05 (2H, –C*H*=N^+^–C*H*–); 8.02–8.43, 7.37–8.04 (11H, –*C*H–C–C*H*–, –O–C_6_*H*_4_–(C=O)–C_6_*H*_5_); 4.19–4.75, 1.45–2.61, 0.59–1.06 (16H, –C*H*_2_–C*H*–C*H*_2_–C*H*–C*H*_2_–C*H*–, –C*H*_2_–C*H*_2_–C*H*_3_).

**1.4. Fabrication of the 1D PC multilayers**

Samples with various concentrations of P2 (from 1.7 wt.% to 4.7 wt.% in 0.3 wt.% increments) were prepared as follows: the **P2** polymer was dissolved in 1-propanol, while 2.5 wt.% **P1** was dissolved in chlorobenzene. Each solution was then passed through a 0.45 μm PTFE syringe filter prior to spin-coating. The polymer solutions were then spin-coated alternately onto a PET film at 2500 rpm for 12 s. Each layer was dried for 1 m to eliminate any remaining solvent, and then exposed to a UVA lamp (652 mJ/cm^2^) prior to the application of the next layer. These processes were repeated to stack a total of 10 layers. A total of 11 films (designated as F1 to F11) were prepared by maintaining the thickness of the **P1** layer and gradually increasing the thickness of the **P2** layer (**Table S1**).

**2. Supplementary Figures**

**Table S1**. The basic details of the eleven 1D PC film samples prepared with various thicknesses.

| Sample | Concentration of solution  (%) | | Spin-coating speed (rpm) | Monolayer thickness (nm) via ellipsometry at 633 nm (nm). | |
| --- | --- | --- | --- | --- | --- |
|  | P1 | P2 |  | P1 | P2 |
| F1 | 2.5 | 1.7 | 2500 | 77 | 77 |
| F2 |  | 2.0 |  |  | 92 |
| F3 |  | 2.3 |  |  | 103 |
| F4 |  | 2.6 |  |  | 124 |
| F5 |  | 2.9 |  |  | 134 |
| F6 |  | 3.2 |  |  | 146 |
| F7 |  | 3.5 |  |  | 161 |
| F8 |  | 3.8 |  |  | 175 |
| F9 |  | 4.1 |  |  | 192 |
| F10 |  | 4.4 |  |  | 207 |
| F11 |  | 4.7 |  |  | 226 |

**
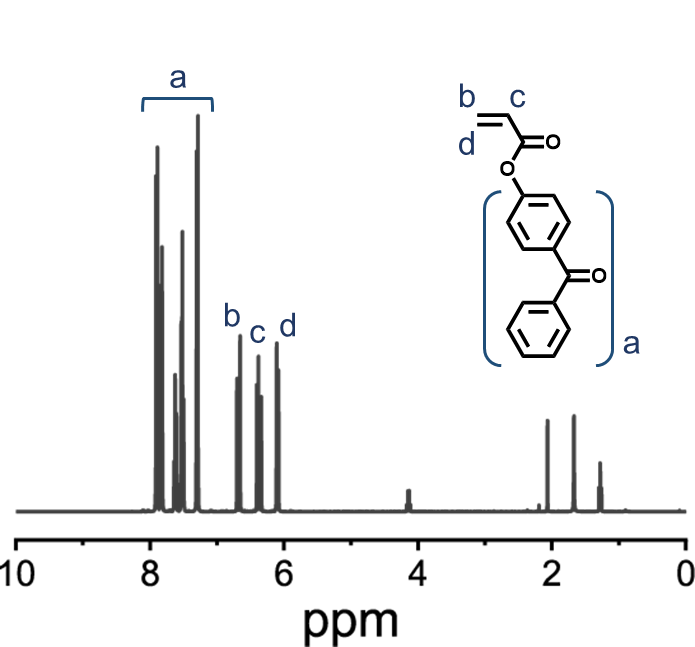
**


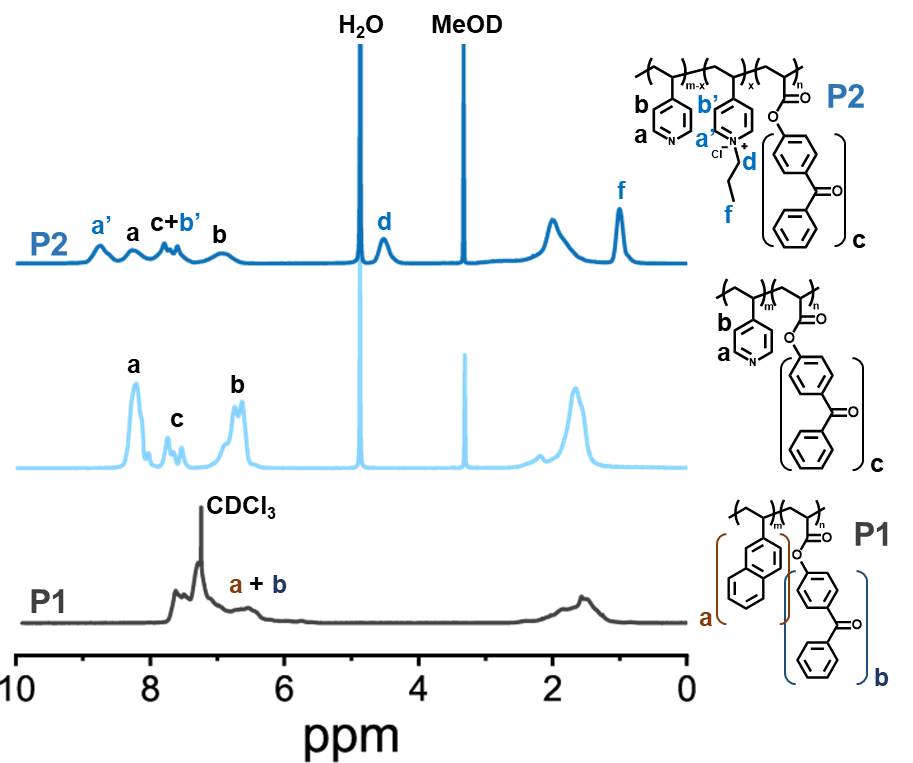


**Figure S1.** ^1^H NMR spectra for synthesis of 4-Benzophenyl acrylate, p(2VN-*co*-BPA) (P1), p(4VP-*co*-BPA), and Quaternized p(4VP-*co*-BPA) (P2).

**
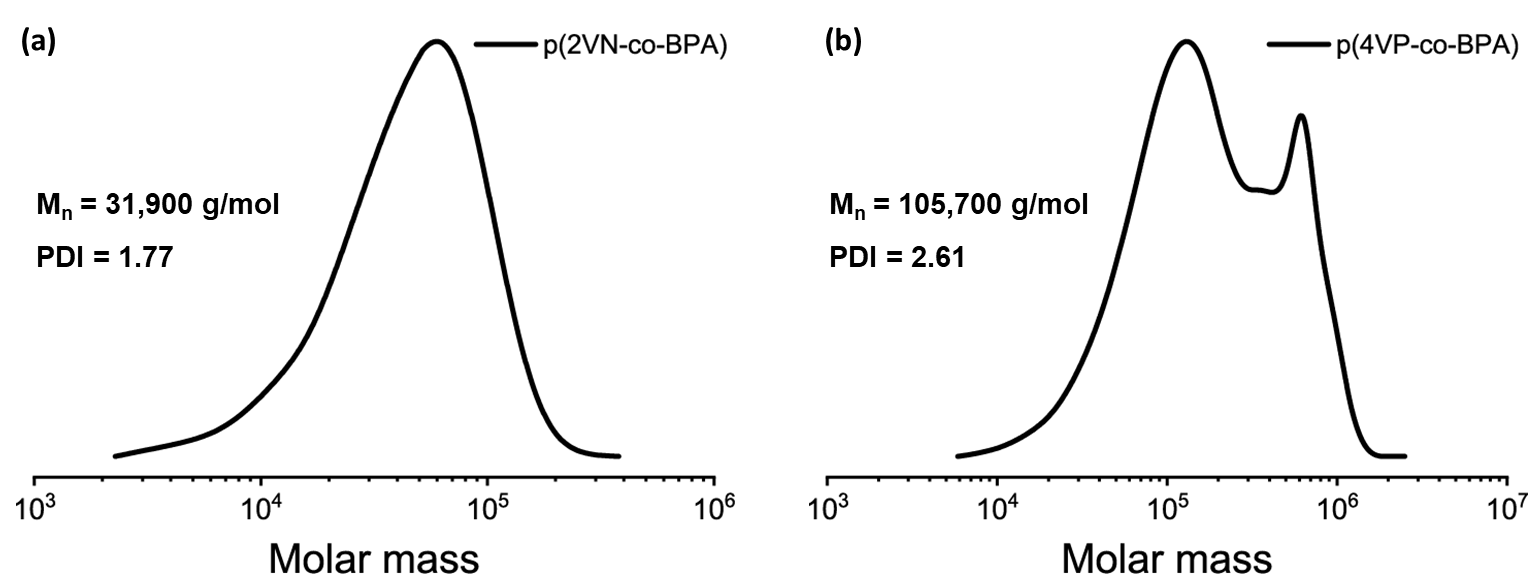
**

**Figure S2.** DMF GPC trace of (a) p(2VN-*co*-BPA), and (b) p(4VP-*co*-BPA).


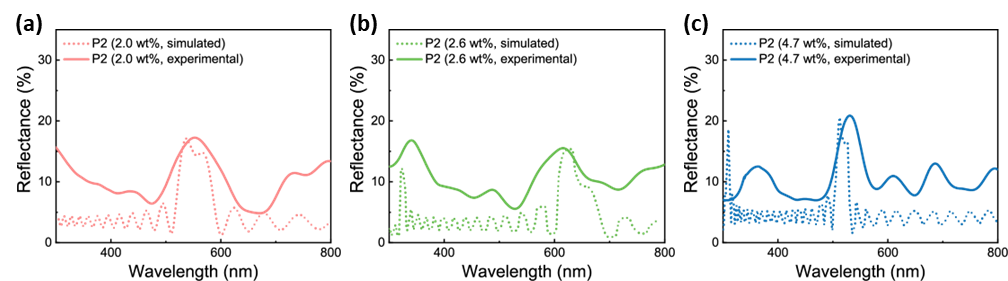


**Figure S3.** Comparing the simulated theoretical reflectance to the experimental reflectance P2 2.0, 2.6 and 4.7 wt%.(under humidity 30%)


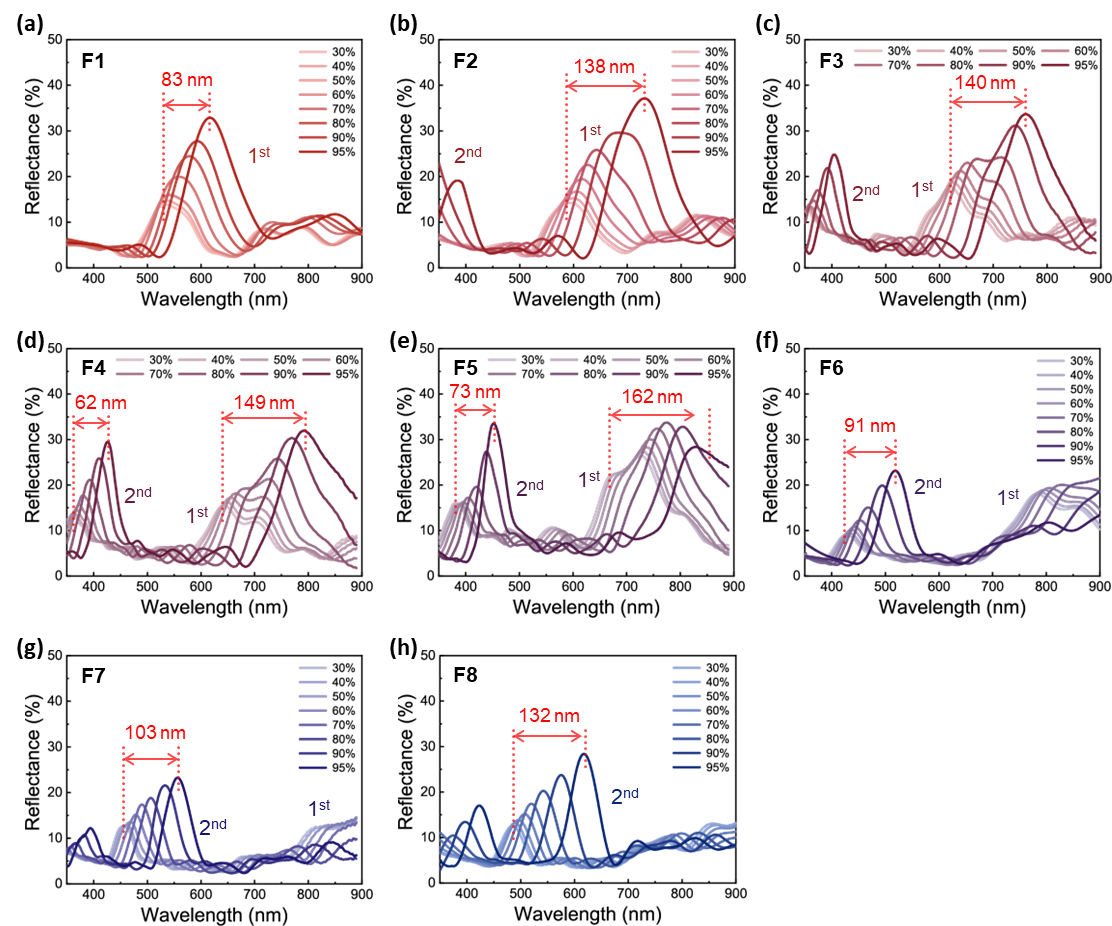


**Figure S4.** The reflectance spectra of samples F1 to F8 at various RH values.


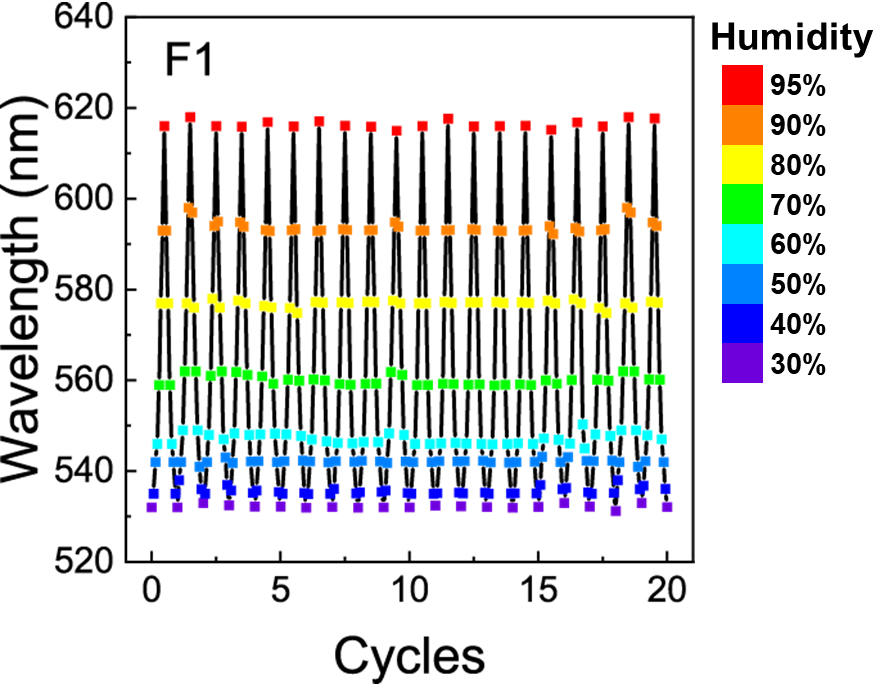


**Figure S5.** Reflectance spectra changes of sample F1 during 20 alternating cycles at various RH values.

**Table S2**. The RGB values of F1 to F11 at RH levels of 30–95%.

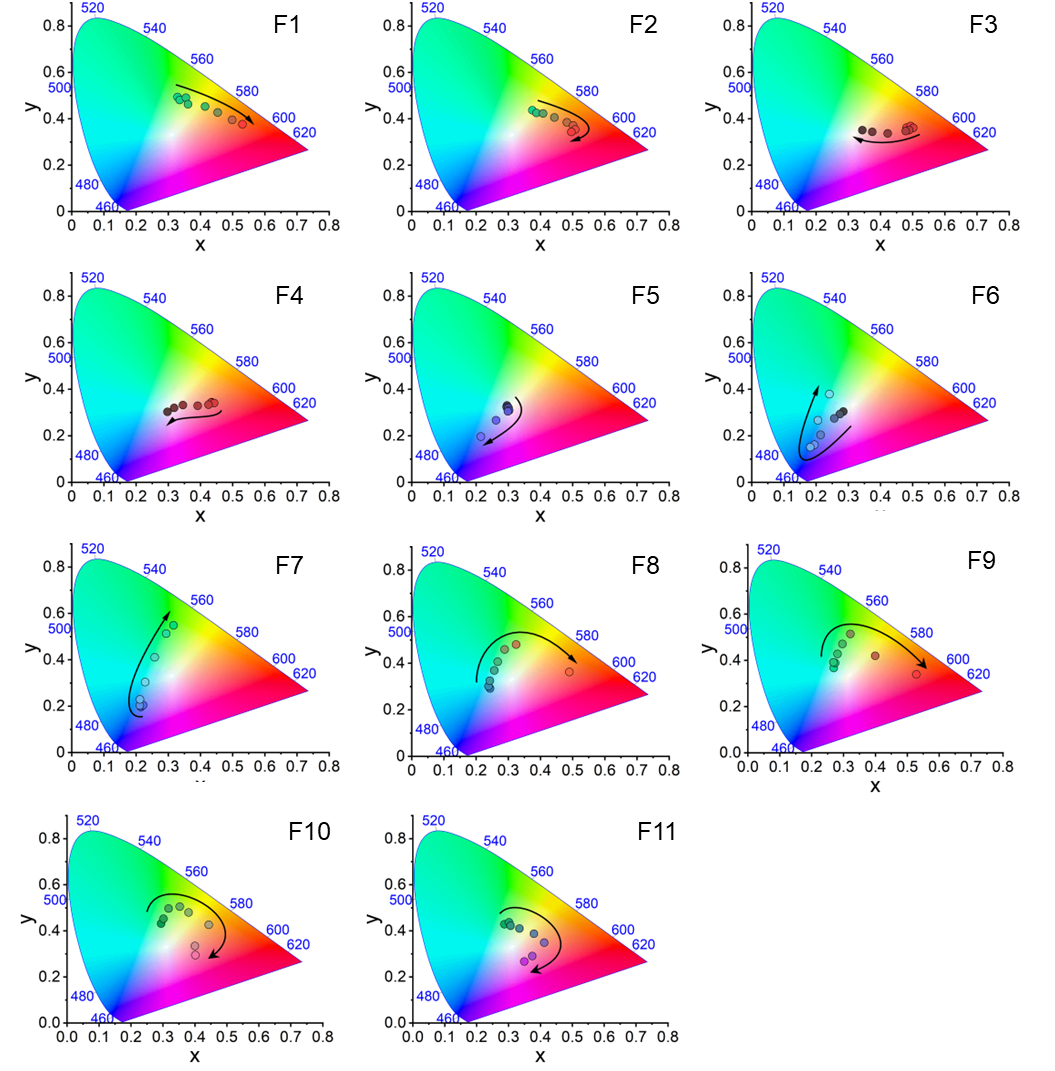


**Figure S6.** The chromaticity diagrams (CIE 1931) of F1 to F11 at RH levels of 30–95%.
